# The thalamic nucleus reuniens orchestrates prefrontal-hippocampal synchrony in memory, emotion, and disease

**DOI:** 10.3389/fnbeh.2026.1885971

**Published:** 2026-07-08

**Authors:** Tuğçe Tuna, Stephen Maren

**Affiliations:** 1 Beckman Institute for Advanced Science and Technology, University of Illinois Urbana-Champaign, Urbana, IL, United States; 2 Department of Psychology, University of Illinois Urbana-Champaign, Champaign, IL, United States

**Keywords:** emotional memory, executive function, network oscillations, neuropsychaitric disorders, nucleus reuniens, prefrontal-hippocampal network, working memory

## Abstract

Decades of literature point to a critical role for prefrontal-hippocampal interactions in emotion and memory. Recent studies in both rodents and humans have revealed that these interactions are coordinated by the midline thalamic nucleus reuniens (RE), a brain region that mediates bidirectional communication between the hippocampus (HPC) and medial prefrontal cortex (mPFC). Here, we aimed to review studies that implicate the RE in memory, emotion, and executive function across a range of behavioral tasks. We also discuss the role the RE may play in the maladaptive cognitive and emotional processes associated with neuropsychiatric and neurological disorders. We conclude that the RE plays a critical role in coordinating oscillations in the mPFC-HPC network to support the flexible and context-dependent expression of memory and emotion.

## 1 Introduction

Interplay between the prefrontal cortex (PFC) and hippocampus (HPC) is critical for many forms of memory, including working memory, spatial memory, episodic memory, and emotional memory (Eichenbaum, 2000, 2017; Griffin, 2015; Jin and Maren, 2015). A major function of this circuit is to promote context-dependent memory retrieval and adaptive behavior that is sensitive to task rules and contingencies (Eichenbaum, 2017; Maren et al., 2013; Dolleman-van der Weel et al., 2019). For instance, a student’s response to a loud ringing bell might differ depending on whether they are in a classroom at the top of the hour (go to the next class) or in the cafeteria during lunch with a whiff of smoke in the air (evacuate). Adaptive behavior depends on integrating contextual information from the internal and external environments with memories of actions appropriate to those contexts and contingencies. This necessitates bidirectional communication between the PFC and the HPC. However, despite direct ventral HPC (vHPC) projections to the medial PFC (mPFC) (Marek et al., 2018; Parent et al., 2010), a few direct dHPC-mPFC connections exist. Similarly, the mPFC has limited direct projections to the HPC [but see Malik et al., 2022 and Rajasethupathy et al., 2015].

**Figure 1:**
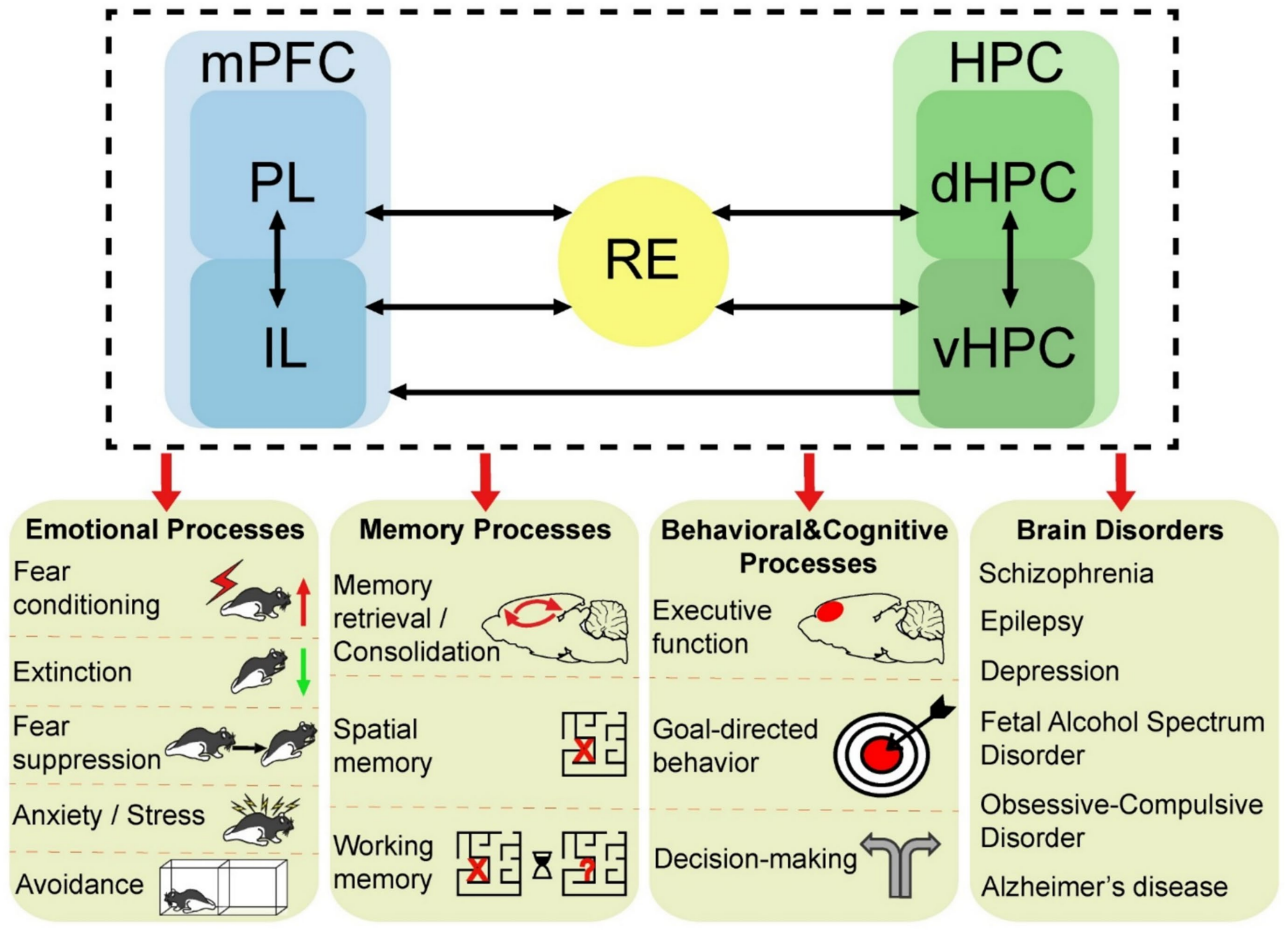
The RE, as a critical hub in the mPFC-HPC network, is involved in emotional, memory, behavioral, and higher-order cognitive processes. Through its bidirectional connections with both the mPFC and HPC, the RE may support these functions by conveying mPFC input to the HPC and HPC input to the mPFC. We propose that the RE dynamically coordinates communication within the mPFC-HPC network by synchronizing oscillatory activity across these regions in a frequency- and task-dependent manner. For example, for context-dependent memory retrieval, the RE may help facilitate mPFC top-down control over HPC-dependent memories by promoting theta-frequency synchrony. Previous research has also implicated the RE in several neuropsychiatric, neurological, and neurodegenerative disorders. mPFC, medial prefrontal cortex; PL, prelimbic cortex; IL, infralimbic cortex; RE, nucleus reuniens; HPC, hippocampus; dHPC, dorsal hippocampus; vHPC, ventral hippocampus.

Despite this asymmetry, extensive anatomical work has revealed that bidirectional mPFC-HPC communication is made possible by the midline thalamic nucleus reuniens (RE) (McKenna and Vertes, 2004; Vertes et al., 2007; Vertes et al., 2006). In recent years, there has been considerable interest in the contribution of the RE to functions of the mPFC and HPC to memory, emotion, and adaptive behavior. There have been several reviews on this topic (Anderson et al., 2016; Cassel et al., 2013; Eichenbaum, 2017; Ferraris et al., 2021; Griffin, 2021; Plas et al., 2024; Dolleman-van der Weel et al., 2019) that underscore the critical role of RE in these processes. Here, we extend this work by arguing that the RE coordinates PFC-HPC interactions to support context-dependent behavior. We first review the anatomical, histochemical, and physiological properties of RE. We then detail the role of the RE across different emotional, spatial, memory, and higher-cognitive tasks. Based on previous findings, we emphasize that modulation of cortical-hippocampal network oscillations by the RE is a critical and shared mechanism that facilitates communication and gives rise to a variety of context-dependent behaviors. We also propose that alterations in RE-mediated cortical-hippocampal communication and network oscillations may underlie disorders such as schizophrenia and epilepsy. Consistent with this, we conclude by reviewing evidence implicating the RE in neuropsychiatric, neurological, and neurodegenerative disorders (Figure 1).

## 2 The nucleus reuniens

The RE is a midline thalamic nucleus that is located above the third ventricle in the rodent brain. Together with the rhomboid nucleus (RH), it is classified as the ventral midline thalamus (Cassel et al., 2013). The RE is not part of the sensory thalamus and therefore is considered a ‘nonspecific’ thalamic nucleus (Vertes et al., 2015a). Despite being studied more heavily in rodents, the RE has been identified in other species, such as humans (Hirai and Jones, 1989; Reeders et al., 2023; Rivera Núñez et al., 2025; Toncray and Krieg, 1946; Tuna et al., 2025a), cats (Room and Groenewegen, 1986; Yanagihara et al., 1985), monkeys (Amaral and Cowan, 1980; DeVito, 1980; Insausti et al., 1987), and ferrets (Herbert, 1963). Below, we review its connections to other brain regions and neurochemical and physiological characteristics, based mostly on data from rodents.

### 2.1 Inputs and outputs

The RE has bidirectional projections with both the mPFC and HPC (Herkenham, 1978; Vertes, 2006; Vertes et al., 2022). As a thalamic nucleus with extensive, bilateral connectivity with both the mPFC and HPC, the RE is positioned to coordinate information flow between the two (Anderson et al., 2016; Eichenbaum, 2017; Ramanathan et al., 2018a; Totty et al., 2023; Tuna et al., 2025a). Approximately 5–10% of RE cells project to both the mPFC and HPC via axonal collaterals (Hoover and Vertes, 2012; Varela et al., 2014). Using anterograde and retrograde tracing methods, Vertes et al. (2007) demonstrated that mPFC fibers form excitatory synapses on proximal dendrites of RE cells that project to the CA1 of HPC. The RE projects more strongly onto the vHPC compared to the dHPC (Hoover and Vertes, 2012; Su and Bentivoglio, 1990; Dolleman-van der Weel and Witter, 1996). RE projections in the HPC target the CA1 region, particularly in the stratum lacunosum moleculare (Vertes et al., 2006; Wouterlood et al., 1990). More recently, Ziółkowska et al. (2024) also showed RE projections to the stratum oriens of the dorsal CA1 in mice, with no projections to the stratum radiatum. HPC-projecting RE neurons are spread more medially and rostrally (Hoover and Vertes, 2012).

In the mPFC, the RE targets both the prelimbic (PL) and infralimbic (IL) cortices, targeting both layers I and V/VI (Vertes et al., 2006). RE neurons that project to the mPFC are largely located both near the midline and laterally in the peri-RE region. Furthermore, they are denser in the caudal levels of the RE (Hoover and Vertes, 2012). The RE forms asymmetrical synapses on both the pyramidal cells and inhibitory interneurons in the mPFC (Cruikshank et al., 2012; Prisco and Vertes, 2006) and the CA1 of HPC (Dolleman-van der Weel and Witter, 2000; Wouterlood et al., 1990).

In addition to the mPFC and HPC, the RE receives input from a diverse set of regions, including the hypothalamus, several other thalamic nuclei, basal forebrain, and the brainstem (Krout et al., 2002; McKenna and Vertes, 2004). However, its projections are limited to limbic cortical areas (Vertes et al., 2006). The RE also has bidirectional connections with the amygdala, entorhinal cortex, and subiculum (Vertes et al., 2022).

### 2.2 Neurochemical properties

Aspartate/glutamate is the primary excitatory neurotransmitter in the RE (Bokor et al., 2002). The RE is devoid of GABAergic inhibitory interneurons (Ottersen and Storm-Mathisen, 1984), despite the presence of GABA receptors (Hallock et al., 2016; Viena et al., 2018; Walsh et al., 2017). However, the thalamic reticular nucleus, consisting of parvalbumin-positive neurons, sends projections to the RE and exerts GABAergic inhibition on RE principal cells. Other non-GABAergic calcium-binding proteins, calretinin and calbindin, are present in RE neurons, and some RE neurons co-express calretinin and calbindin (Arai et al., 1994; Bokor et al., 2002; Viena et al., 2020). Importantly, Viena et al. (2020) demonstrated that RE neurons projecting to both mPFC and HPC do not express either calbindin or calretinin. Therefore, a more detailed exploration of the histochemical profiles of RE projection neurons is necessary. Interestingly, this pattern is very similar to mPFC- and HPC-projecting paraventricular nucleus neurons, with the dual-projecting neurons expressing neither calbindin nor calretinin (Viena et al., 2022), which may be in line with views suggesting a functionally unitary limbic thalamus (d' Vasconcelos and Cassel, 2015; Vertes et al., 2015b). Ogundele et al. (2017) showed that dopaminergic neurons are also present in the RE, which project to the paraventricular nucleus. They proposed that dopaminergic cells in the RE may help regulate pre-autonomic sympathetic events, given their connections to the paraventricular nucleus and rostral ventrolateral medulla. However, the function of these dopaminergic neurons is yet to be determined.

### 2.3 Physiological characteristics

Studies examining the physiological properties of RE neurons are scarce, although researchers have investigated physiological responses in the mPFC and HPC evoked by the RE. Here, we provide a review for both. Using patch clamp recordings in mouse brain slices, Walsh et al. (2017) provided a detailed characterization of the intrinsic firing properties of rostrally-located RE neurons. They reported that the majority of RE neurons (88%) fire spontaneously during whole-cell recording mode. These cells had a resting membrane potential of −63.7 ± 0.6 mV, lower than the cells that were silent during the same recording mode (12%; −70.6 ± 3.2 mV). Overall, more than 70% of the recorded RE neurons exhibited constant firing at 2–20 Hz. Authors observed that afterhyperpolarization is critical in determining the firing rate. Both square-wave current injections and sufficiently large EPSP-like currents led to firing in the majority of RE neurons, with an initial bursting pattern. Interestingly, Walsh et al. (2017) observed that, after blocking GABAergic and glutamatergic synaptic transmission, RE neurons fired at approximately 8 Hz, suggesting that RE neurons may generate theta oscillations intrinsically.

Lara-Vásquez et al. (2016) recorded activity from midline thalamic neurons, including some in the RE, in anesthetized mice. Among the recorded RE neurons, some co-expressed calbindin and calretinin, some expressed only calbindin or calretinin, and some expressed neither. Calretinin-expressing neurons revealed consistently lower spontaneous firing and mean firing rates compared to calretinin-negative neurons. However, calretinin-positive neurons were more likely to burst, despite being more silent overall. Lara-Vásquez et al. (2016) then recorded single-unit activity from the midline thalamus while simultaneously recording the local field activity from the dHPC. Calbindin expression did not predict differential firing during hippocampal theta. However, calretinin expression was related to differential firing patterns in response to hippocampal theta and sharp wave ripples. Specifically, calretinin-positive neurons did not change their firing rates during hippocampal theta oscillations compared to calretinin-negative neurons. Calretinin-negative neurons were more active during both theta and non-theta states, despite only a small number showing phase coupling to the oscillatory cycle and no phase preference during theta. However, an opposite pattern was observed for sharp wave ripples: while calretinin-positive neurons were modulated by sharp wave ripples, calretinin-negative neurons did not change their firing during these states. Overall, these results may provide insights into the firing patterns of RE calbindin- and calretinin-expressing neurons within the larger midline thalamic neuronal population.

Zimmerman and Grace (2018) reported tonic and burst firing patterns in the RE in rats under anesthesia. Most RE neurons showed both burst and tonic firing with a mean firing rate of 2.16 ± 0.36 Hz. A smaller proportion of RE neurons showed higher firing rates between 4 and 12 Hz. Hauer et al. (2019) recorded from single RE neurons in urethane-anesthetized rats during forebrain theta and slow oscillation states. They demonstrated slow rhythmic activity in RE neurons during slow oscillations, which were phase-locked to this ongoing forebrain state. During theta states, however, RE neurons were more active and fired significantly more spikes. Furthermore, tonic firing was observed during theta states. Using multi-unit activity recordings, Hauer et al. (2019) confirmed slow, rhythmic burst firing during slow oscillations and higher-frequency and tonic firing patterns during theta states.

Zimmerman and Grace (2018) revealed that the firing of RE neurons is modulated by the IL region of the mPFC. Inhibiting IL activity with TTX infusions did not change the firing rate or the number of spontaneously active RE neurons. However, it decreased both burst firing and the number of spikes within a burst. On the other hand, electrical stimulation of the IL led to an initial cessation of firing in RE neurons. Then, it either increased burst firing or led to a complete cessation of firing. Since IL input to the RE is glutamatergic, this effect is likely induced by IL projection to the thalamic reticular nucleus, which has GABAergic projections to the RE. Conversely, reticular nucleus inhibition with muscimol decreased the number of spontaneously active RE neurons. Zimmerman and Grace (2018) examined the effect of silencing direct IL input to the RE by chemogenetically inhibiting IL terminals in the RE. This increased the percentage of burst spike firing in a subset of RE neurons. Therefore, cortical input may differentially affect RE firing properties depending on whether it is direct or indirect.

Bertram and Zhang (1999) recorded from the HPC in urethane-anesthetized rats while electrically stimulating the RE/RH, and reported EPSPs and spikes in the CA1. These responses were monosynaptic as indicated by their latencies. Confirming this finding, Hauer et al. (2019) showed that optogenetic stimulation of the RE results in evoked potentials in the lacunosum moleculare layer of the CA1. Paired-pulse stimulation of the mPFC similarly evoked potentials in the CA1, although with a longer latency, suggesting that the RE may be the intermediary structure between the mPFC and HPC. Indeed, chemogenetic silencing of the RE blocked mPFC stimulation-evoked potentials in the HPC, and single- and multi-unit recordings from the RE during mPFC stimulation caused orthodromic excitation in RE neurons. Moreover, chemogenetic silencing of the RE significantly decreased mPFC-HPC slow oscillatory coupling. Goswamee et al. (2021) optogenetically activated RE afferents and recorded synaptic responses in the CA1 region of mice. They revealed that, following theta-burst and gamma stimulation, CA1 pyramidal cells show small EPSPs in 9/10 recorded cells. However, RE terminal stimulation led to action potential firing in only 1/10 of cells. Notably, these recordings were performed in *ex vivo* hippocampal slices, and *in vivo* responses may differ. Goswamee et al. (2021) then performed voltage clamp experiments to examine IPSC and EPSC responses in CA1 principal cells following RE terminal stimulation. Of the responsive neurons, most responded to both IPSCs and EPSCs, with some principal neurons showing only IPSCs and some showing only EPSCs. Hence, stimulation of the RE results in both inhibition and excitation in CA1 principal neurons. EPSCs were due to monosynaptic excitation of CA1 principal neurons and were mediated by AMPA receptors, whereas IPSCs were polysynaptic and GABAAR-mediated. Inspecting the morphology and firing properties of recorded neurons, Goswamee et al. (2021) concluded that the origin of inhibitory currents was due to the recruitment of neuropeptide Y-containing putative neurogliaform cells in the CA1. RE stimulation activated half of the recorded interneurons, and stimulation combined with the selective silencing of neuropeptide Y-containing neurons decreased IPSCs.

Conversely, using tracing, ex vivo optogenetics, and electrophysiological recordings in mice and rats, Andrianova et al. (2025) recently showed that the RE does not innervate pyramidal cells in the HPC. Optogenetic stimulation of the RE did not elicit postsynaptic EPSCs in dCA1 or vCA1 pyramidal neurons. The authors emphasized the differences between their results and those of Goswamee et al. (2021), suggesting that the recordings by Goswamee et al. (2021) may have occurred in the subiculum rather than the CA1. However, Andrianova et al. (2025) observed evoked postsynaptic responses in neurogliaform cells in these same recordings. The RE targeted pyramidal cells in the prosubiculum and subiculum instead, although with a low synaptic strength. Therefore, the authors concluded that the RE is unlikely to elicit spikes in hippocampal pyramidal neurons due to the absence of connections or subthreshold connections. These results by Andrianova et al. (2025) are in line with earlier findings by Dolleman-van der Weel et al. (1997), who showed that, in anesthetized rats, RE input to the CA1 cannot discharge pyramidal neurons despite activating local interneurons in the stratum oriens/alveus and radiatum. They concluded that RE leads to subthreshold depolarization in pyramidal cells while evoking suprathreshold excitation in inhibitory interneurons. Moreover, Leprince et al. (2023) revealed that in mouse pups, the ventromedial thalamus, including the RE, directly activates GABAergic interneurons rather than principal cells. In line with these findings, Sun et al. (2014) showed only weak RE-to-CA1 projections.

The HPC → mPFC direction of information flow, with the RE as intermediary, is less well studied. Basha et al. (2025) showed both orthodromic and antidromic responses in mPFC neurons following RE electrical stimulation in anesthetized cats. Another recent report by Vantomme et al. (2025) has demonstrated both monosynaptic excitatory and disynaptic feedforward inhibitory responses in RE → mPFC synapses in mice. RE stimulation causes prolonged excitation in mPFC neurons, which may act to amplify hippocampal signal to the mPFC, through the HPC → RE → mPFC network.

Overall, these findings reveal inconsistencies, particularly regarding RE’s ability to evoke firing in HPC principal cells. Differences in recording methods, tools, locations, and stimulation parameters might account for contradictory findings. More recording studies are needed to elucidate the effects of the RE input on spike firing in different types of hippocampal neurons. HPC → RE → mPFC pathway, which is relatively overlooked compared to the mPFC→RE → HPC pathway, should also be examined in more detail, including its physiological properties. Finally, most of these findings come from adult male rats or mice. Future work should include females to look for potential sex differences.

## 3 RE in affective and memory-guided behavior

### 3.1 Emotional memory

#### 3.1.1 Fear and extinction memories

Insofar as both the mPFC and HPC are critically involved in fear and extinction memories (Kalisch et al., 2006; Maren, 2001; Milad et al., 2007; Phillips and LeDoux, 1992), researchers have turned their attention to the RE as a bridge between the two. Ramanathan et al. (2018b) pharmacologically inactivated the RE either before contextual fear conditioning or before its retrieval. RE inactivation prior to conditioning impaired contextual freezing, whereas inactivation prior to retrieval increased fear generalization to a novel context. These results are consistent with previous findings by Xu and Südhof (2013), who demonstrated that silencing the RE increases fear memory generalization, whereas activating the RE decreases this generalization. They proposed that the RE contributes to memory specificity in a given context by processing cortical input to the HPC. Interestingly, Ramanathan et al. (2018b) revealed that contextual fear conditioning deficits resulting from RE inactivation disappear when the retrieval test is conducted under RE inactivation. In other words, deficits in contextual fear conditioning after RE inactivation were state-dependent. Moreover, hippocampal NMDA antagonism did not affect contextual fear conditioning in rats trained after RE inactivation, suggesting that contextual fear acquired in the absence of RE is HPC-independent. Together, these results demonstrate that when the RE is offline, contextual memories are formed independently of the HPC, and therefore, can be imprecise and prone to generalization. However, when the RE is intact, it can engage the HPC to form precise contextual memories.

Consistent with these findings, Lin et al. (2020) showed that the RE is also required for the acquisition of trace fear memories, and the retrieval of trace fear acquired without the RE was state-dependent. Ratigan et al. (2023) silenced RE-to-dCA1 projections in mice and revealed an extinction impairment after a virtual reality contextual fear conditioning procedure. They reported that inhibiting this pathway leads to higher freezing levels, fear generalization, and delayed extinction learning. Ziółkowska et al. (2024) also examined RE-to-dCA1 projections and revealed that the excitatory synapses RE forms in the CA1 undergo structural changes with contextual fear conditioning. Chemogenetic inhibition of this pathway impaired both contextual fear and structural changes in synapses in the lacunosum moleculare layer. Overall, these results show that RE is involved in the acquisition, extinction, and retrieval of contextual fear memories.

Ramanathan et al. (2018b) also showed that pharmacological inactivation of the RE spares auditory fear conditioning, indicating that cued fear memories can be acquired when the RE is offline. However, examination of the role of RE in the acquisition and retrieval of cued fear extinction by Ramanathan et al. (2018a) revealed that the RE is critically involved in both processes. Muscimol infusions into the RE before extinction impaired the acquisition of extinction. When tested for retrieval the next day, animals also showed significantly greater freezing compared to control animals. Similarly, rats that underwent a drug-free extinction session but received muscimol before the extinction retrieval test showed significantly greater freezing than controls. Extinction training and extinction retrieval increased the immediate early gene (IEG) c-Fos expression in the RE compared to home-cage controls. Furthermore, single-unit recordings revealed increased CS-evoked firing during extinction retrieval. Totty et al. (2023) confirmed the relapse of fear with pharmacological inactivation of the RE prior to extinction retrieval. They reported decreases in c-Fos expression in the IL and PL cortices as well as the vHPC, suggesting that RE activity may recruit the mPFC and HPC during extinction retrieval. RE activity may be critical in inhibiting conditioned fear by recruiting the mPFC and HPC, whereas in the absence of the RE, freezing cannot be suppressed. The results mentioned above by Ratigan et al. (2023) and Ziółkowska et al. (2024) are also in line with this.

More recently, Hassell et al. (2025) has coined the term ‘circuit-induced relapse’ to explain the relapse of fear after extinction when the RE is inactivated. They showed that HPC-dependent contextual fear memories are required for extinction retrieval deficits (i.e., relapse) associated with RE inactivation. Specifically, they showed that APV infusions in the dHPC prevent the acquisition of contextual fear memories and block the circuit-induced relapse caused by intra-RE muscimol infusions. Moreover, the relapse of extinguished fear in response to an auditory CS could be elicited by chemogenetic activation of hippocampal fear engrams. These authors proposed that the RE contributes to context-dependent fear extinction by suppressing the retrieval of the fear engram in the extinction context. Additional data suggested that it might also participate in retrieving extinction engrams in the HPC to further suppress fear. Consistent with this, Lacagnina et al. (2019) showed distinct fear and extinction engrams in the HPC.

Which upstream brain region provides the RE with the signal necessary to recruit the HPC for suppressing conditioned freezing and facilitating extinction memory retrieval? Ramanathan et al. (2018a) examined the possibility that mPFC projections to the RE are critical for their role in extinction acquisition and retrieval. They showed that chemogenetic silencing of RE-projecting mPFC neurons causes extinction retrieval deficits. Chemogenetic silencing of mPFC terminals in the RE similarly impaired extinction retrieval. These findings reveal that the mPFC-to-RE projection modulates extinction memories and that the mPFC may exert top-down control over fear suppression via its projections to the RE, thereby recruiting the HPC.

A recent report by Tuna et al. (2025b) provided a cross-species longitudinal examination of changes in RE activity as rats and healthy human participants underwent cued fear conditioning, extinction, and extinction retrieval. Both the BOLD signal in humans and the calcium signal in rats revealed that RE acquires associative value for the CS: CS-evoked activity increases during conditioning as CSs are paired with shock USs, and then decreases with extinction training. Furthermore, both BOLD and calcium signals in humans and rats, respectively, were low during extinction retrieval. This latter finding is at odds with previously reported increases in c-Fos and CS-evoked firing (Ramanathan et al., 2018a) and with a role for the RE in suppressing conditioned fear. However, Tuna et al. (2025b) observed that a subset of CS-responsive neurons recorded using electrophysiological procedures in behaving rats indeed show a robust CS-evoked firing during extinction retrieval. Furthermore, these recordings revealed that RE contains distinct subpopulations of neurons that preferentially fire during a high-fear state (early extinction; fear cells) and during a low-fear state (late extinction; extinction cells). These results are consistent with a fear-suppression role for the RE. RE extinction cells may play a role in suppressing conditioned freezing.

Mochizuki et al. (2025) recorded calcium activity from RE neurons projecting to the vmPFC in male mice. They found that vmPFC-projecting RE neurons exhibit increased responses to a CS paired with foot shock, but not to a CS that is not shock-paired. During extinction learning, Mochizuki et al. (2025) observed a biphasic response to the shock-paired CS, with initially increased activity at the onset of CS and suppressed activity at the offset. During extinction retrieval, response to the shock-paired CS decreased to the levels of the shock-unpaired CS. Optogenetic inactivation of the vmPFC-projecting RE neurons during extinction did not affect freezing behavior. There was also no effect on freezing behavior during the extinction retrieval test. Therefore, mPFC input to HPC via the RE, but not the input in the opposite direction from the RE to mPFC, may be crucial for the suppression of fear or freezing behavior.

Silva et al. (2021) examined the role of RE projections to the BLA on the extinction of fear memories and found that these projections are recruited for the extinction of 30-day-old (remote), but not 1-day-old (recent), fear memories. Chemogenetic inhibition and excitation of the RE or RE-to-BLA projections impaired and facilitated remote fear memory extinction, respectively, revealing a bidirectional modulation of remote fear extinction by the RE. Venkataraman et al. (2021) showed that zona incerta-to-RE projections impact extinction memories. Optogenetic stimulation of both GABAergic and dopaminergic zona incerta-to-RE terminals enhanced extinction retrieval. Cheung et al. (2024) revealed that extinction learning and retrieval can be facilitated by optogenetic activation of vHPC-to-lateral septum projections that receive input from the RE. Finally, Tomaszewski et al. (2026) examined the role of RE-to-medial septum projections in extinction and found that these projections modulate extinction memory on remote (30-day) but not recent (1-day) time points. These results once again show the importance of RE in extinction processes, urging us to characterize other networks that may be involved in extinction learning and memory, beyond the mPFC-HPC network.

#### 3.1.2 Freezing suppression

Recent work suggests that the RE is involved in suppressing freezing behavior. Silva et al. (2021) showed that calcium signals from the RE and RE-to-BLA projections are correlated with freezing behavior, with increases in activity preceding the cessation of freezing behavior. Moreover, closed-loop photo-inhibition of the RE prolonged freezing bouts, whereas photo-stimulation shortened them, suggesting that RE contributes to the cessation of freezing behavior and may signal safety to the BLA. These results were extended by Tuna et al. (2025b). Using fiber-photometric recordings in the RE, Tuna et al. (2025b) showed that spontaneous calcium activity of the RE tracks defensive state transitions from freezing to activity in an extinguished context, but not a neutral context prior to conditioning. Specifically, freezing initiation was associated with a decrease in RE activity, whereas freezing termination was associated with an increase in RE activity. The increase in RE activity preceded freezing termination, suggesting that RE may be actively suppressing conditioned freezing. Tuna et al. (2025b) revealed similar activity patterns in single RE neurons: their spontaneous firing tracked behavioral transitions with overall higher firing during movement and lower firing during freezing. However, some neurons showed the opposite firing pattern.

Furthermore, Ratigan et al. (2023) reported that RE activity is tuned to conditioned freezing behavior. They performed two-photon imaging of RE terminals in dCA1 during the retrieval of a contextual fear memory, which showed that calcium activity increases during bouts of freezing and decreases during running. Moreover, this pattern of activity was present only during fearful freezing epochs, and not non-fearful freezing epochs prior to conditioning, and it decreased with extinction training. Ratigan et al. (2023) concluded that the RE-CA1 projection suppresses fear by disrupting contextual fear memory in the HPC. Notably, this activity pattern is the opposite of what is observed by Silva et al. (2021) and Tuna et al. (2025b). However, Tuna et al. (2025b) also observed increased RE neuronal firing during freezing. They speculated that these RE neurons may be the ones projecting to the HPC, which would confirm Ratigan et al. (2023) results. Contradictory findings may be explained in part by differences in species, conditioning protocols, and memory age in these studies. Local axonal signals may also be decoupled from the somatic activity. These findings may potentially reflect functional heterogeneity of the RE with its projection-specific roles in regulating defensive behavior. The RE may contain distinct subpopulations that are recruited at different phases of defensive state transitions and that transmit different signals to downstream targets. For instance, RE-to-BLA projection may contain a safety (freezing termination) signal. Conversely, RE-to-dHPC projection may be recruited during ongoing freezing epochs to suppress contextual fear memories in the HPC. Thus, RE-to-dHPC projection may be actively involved in freezing suppression rather than promoting it. Finally, Moscarello (2020) proposed a freezing suppression role for the vmPFC-to-RE projections following signaled active avoidance training. The recruitment of this pathway to suppress freezing is adaptive, as proactive avoidance responses can be engaged in the absence of freezing. Collectively, these findings demonstrate that RE activity is crucial for modulating fearful freezing states. The RE may serve as a hub coordinating transitions between defensive states by routing prefrontal signals to hippocampal and amygdalar targets in a projection- and state-dependent manner.

#### 3.1.3 Avoidance

Flämig and Klingberg (1978) showed that RE lesions do not affect the conditioned avoidance responses in a Y-maze. However, there is some evidence pointing to a role for RE in avoidance. Davoodi et al. (2011) examined the role of the RE in the acquisition, consolidation, and retrieval of passive avoidance. They demonstrated that RE inactivation with local tetracaine infusions 5 min before the passive avoidance task does not affect acquisition. However, this pre-training inactivation of the RE impaired retention. Tetracaine-induced inactivation of the RE, which occurred at 5 min, but not at 90 or 360 min, after avoidance training, impaired consolidation. Moscarello (2020) demonstrated that the vmPFC projects to the RE following training in a signaled active avoidance task, thereby suppressing freezing behavior. Chemogenetic inactivation of the vmPFC after two-way signaled active avoidance training and 20 min before the CS test increased freezing to CSs. Chemogenetic inactivation of vmPFC terminals in the RE and muscimol inactivation of the RE similarly increased freezing to CSs. Moscarello (2020) proposed that the vmPFC-to-RE pathway may be a shared mechanism underlying the suppression of conditioned freezing for different memory tasks. Linley et al. (2021) showed that muscimol inactivation of the RE/RH decreases time spent in the open arms of an elevated plus maze, suggesting avoidance behavior. Furthermore, exposing rats to the elevated plus maze increased Fos levels in the RE/RH. The authors proposed that RE/RH may act to promote adaptive behaviors in anxiogenic situations, and their inactivation leads to maladaptive coping responses. Overall, the RE may be involved in adaptive avoidance behavior. However, more studies are necessary to further understand the role of the RE in avoidance behavior.

#### 3.1.4 Anxiety

Linley et al. (2021) demonstrated that elevated plus maze exposure increases c-Fos expression in the RE. Guo et al. (2024) reported RE projections to the lateral septum GABAergic neurons as an important player in chronic itch-induced anxiety in mice. Chronic itch induces plastic changes in this pathway, and the inhibition of this pathway reduced chronic itch-induced anxiety-like behaviors in open-field and elevated plus maze tasks. However, this pathway was not involved in restraint-stress-induced anxiety-like behaviors. Goh et al. (2025) showed that optogenetic stimulation of the RE may evoke anxiety. In a place-preference test, rats spent less time in the stimulation-paired compartment, indicating that RE stimulation may be aversive. Furthermore, RE stimulation increased time spent at the periphery rather than the center in an open field test, indicative of increased thigmotaxis behavior and anxiety. In line with Linley et al. (2021) results, Goh et al. (2025) found that RE stimulation decreases the exploration of open arms in an elevated plus maze. Overall, Goh et al. (2025) proposed that RE photo-stimulation is aversive and anxiogenic. Xu et al. (2025) examined the one-trial tolerance phenomenon observed in the elevated plus maze, in which one exposure to the maze prevents open arm entries in the following exposures. The authors showed that MK-801, an NMDA receptor antagonist, can prevent this phenomenon and increase Fos expression in the RE. Furthermore, chemogenetically inactivating the RE blocked MK-801 effects on one-trial tolerance. Rivera Núñez et al. (2025) provided evidence for the involvement of the RE in anxiety in humans. Adolescents with varying degrees of anxiety levels were shown negative, positive, and neutral images as part of an emotional similarity task while in an MRI scanner. In the test session, after 12 h, they were shown old images (targets), new images (foils), and images slightly different from the old ones (lures), and were asked to indicate whether each image was old or new. RE activity was elevated for negative lures compared to neutral-lure false alarms during the test, revealing that the RE modulates negative overgeneralization. Furthermore, the RE showed increased functional coupling with the HPC for negative compared to neutral false alarms at test. These results suggest that RE is involved in anxiety in both rodents and humans; however, further studies are needed to reveal its complete role.

### 3.2 Spatial memory

The RE has been shown to have spatially tuned neurons. Jankowski et al. (2014) identified head-direction cells in the RE in freely moving rats. The activity of these cells was not affected by changing the lighting or shape of the recording environment. Furthermore, these cells did not remap with time or sleep/wake state but were modulated by direction. Later, Jankowski et al. (2015) identified RE neurons with properties similar to hippocampal and parahippocampal place cells and perimeter/border cells. Place cells in the RE exhibited larger receptive fields and lower spatial information compared to those in the anteromedial and parataenial thalamic nuclei. Perimeter/border cell firing was observed from the first minute of exposure to a circular environment and was constant throughout recording. These cells fired when rats were close to the boundaries of the recording environment. These findings by Jankowski et al. (2015) suggest a role for RE in the spatial representation of the environment. Importantly, Cholvin et al. (2018) showed that the RE is involved in hippocampal place cell firing and field stability. In this work, electrophysiological recordings from CA1 place cells took place in familiar and novel environments after RE/RH lesions. RE/RH lesions did not affect remapping of hippocampal place cells in novel environments. However, compared to sham surgeries, RE/RH lesions reduced the spatial coherence of hippocampal place cells in novel environments with noisier spatial firing patterns. There was an overall impairment in field stability at the baseline and in the familiar environment after exposure to novel environments. Field stability was further impaired with exposure to novel environments.

Davoodi et al. (2009) studied the role of the RE in the acquisition, consolidation, and recall of spatial reference memory using the Morris water maze (MWM), an HPC-dependent task. Tetracaine-induced inactivation of the RE before the MWM impaired the acquisition and increased escape latencies compared to saline controls. However, both groups performed comparably on the recall test. Tetracaine infusions immediately after the training decreased the time spent in the target quadrant during the recall test the next day, suggesting impairments in consolidation. Tetracaine infusions administered immediately before the recall test also decreased time spent in the target quadrant, indicating memory retrieval impairments. Conversely, Dolleman-van der Weel et al. (2009) showed that RE-lesioned rats learn and recall the location of the platform in a water maze task. These distinct findings may be explained by the type of RE inactivation used. Reversible inactivation of the RE right before a test may impair acquisition or recall by disrupting ongoing RE-mediated communication with the HPC. Whereas permanent lesions may allow for compensatory circuits to be involved. RE/RH lesions were shown to produce deficits in the radial arm maze, a common task of spatial memory affected by both mPFC and HPC lesions (Hembrook and Mair, 2011). Loureiro et al. (2012) demonstrated that RE is involved in the consolidation of long-term spatial memories. Interestingly, Huang et al. (2021) reported projections from the retinal ganglion cells to the lateral geniculate nucleus (LGN), which then innervate the RE. This retina-LGN-RE pathway mediated the facilitation of spatial memory with bright light treatment. These results suggest a contribution of the RE in spatial processing as well as spatial memories.

### 3.3 Working memory

RE is heavily implicated in working memory tasks in which animals must temporarily hold onto task-relevant information that will guide their decisions. This involvement is tied to its role in facilitating communication between the mPFC and the HPC, both of which are involved in working memory (Griffin, 2015; Griffin, 2021). Consistent with this, Hembrook et al. (2012) reported impairments in the mPFC- and HPC-dependent delayed nonmatching-to-position task, but not in HPC-dependent varying choice radial maze delayed nonmatching task, following low-dose RE muscimol inactivation. Similarly, RE muscimol inactivation impaired working memory-dependent, but not working memory-independent, performances on a conditional discrimination task (Hallock et al., 2013). RE inactivation in a spatial working memory-dependent delayed alternation task also impaired performance compared with pre-infusion baseline and saline-infused controls (Hallock et al., 2016). Davoodi et al. (2009) inactivated the RE with tetracaine either right before or after the last training session in the MWM. Animals tested 75 min after tetracaine infusions showed significantly longer escape latencies than saline-treated controls, suggesting impaired memory retention. Optogenetic inactivation of the RE in a T-maze delayed nonmatch-to-position task, a spatial working memory-dependent task, was shown to impair acquisition (when opto-stimulation was delivered during the sample phase) but not retention (delay phase) or retrieval (choice phase). Decreased choice accuracy during the sample phase recovered when the opto-stimulation ceased, suggesting an online disruption of RE function rather than a permanent learning deficit (Maisson et al., 2018). Boch et al. (2022), using the double H maze, also examined the stage at which the RE is involved in spatial working memory. RE muscimol or chemogenetic inactivation did not affect performance in the maze during acquisition, retention, or retrieval. These contradictory findings may be partly explained by differences in tasks (T-maze vs. double-H maze), manipulation types, and delay periods. Furthermore, RE’s involvement may vary with the extent to which coordinated mPFC-HPC interaction is required. The RE may be more heavily involved in spatial working memory tasks when animals must encode and use trial-specific information through coordinated mPFC-HPC processing.

Morvan et al. (2022) revealed a positive correlation between the activity level in the RE, as shown by c-Fos, and delayed (6 h after training) working memory performance in a double H maze. Previous evidence suggests that the mPFC and HPC interaction increases in working memory tasks with longer delays (Churchwell and Kesner, 2011). Therefore, longer delays may involve the RE for mPFC-HPC communication. In line with this, Layfield et al. (2015) demonstrated that RE muscimol inactivation in a continuous alternation task does not impair performance. However, in a delayed alternation task, RE inactivation with all muscimol doses disrupted performance on the long delay (30 s) but not the short delay (5 s), which was disrupted only with the highest muscimol dose. Viena et al. (2018) reported impaired spatial working memory in a delayed nonmatch to sample spatial alternation T-maze task following RE muscimol inactivation for three delay periods: 30, 60, and 120 s. Procaine inactivation, on the other hand, decreased choice accuracy only for the 120 s delay. Despite its role in working memory, RE’s involvement may change with delay periods and tasks. For more detailed reviews on RE’s involvement in spatial working memory, see Griffin (2015); Griffin (2021).

### 3.4 RE and memory persistence, consolidation, and reconsolidation

Memory consolidation involves offline replay of newly acquired memories, which is marked by a slow oscillatory activity coupling between the mPFC and HPC (Buzsáki, 1998; Diekelmann and Born, 2010; Klinzing et al., 2019). There is evidence that the RE facilitates this slow oscillatory mPFC-HPC coupling. The RE facilitates information exchange within the prefrontal-hippocampal network during slow-wave sleep, and RE inactivation impairs the mPFC-HPC slow oscillatory synchrony in anesthetized rats. Therefore, the RE may be involved in memory consolidation processes (Angulo-Garcia et al., 2020; Ferraris et al., 2021; Ferraris et al., 2018; Varela and Wilson, 2020). A recent report by Lee et al. (2025) also showed that the RE is part of the homeostatic sleep circuit: a subset of RE neurons was activated by sleep need, and optogenetic activation of them was sufficient to induce deep recovery sleep.

Mei et al. (2018) trained rats to collect rewards in a crossword-like maze. At the end of each training session, RE was reversibly inactivated with muscimol. RE inactivation did not affect spatial task performance or memory retention after 20 days. However, RE inactivation after training and prior to testing impaired the spatial memory retrieval, suggesting a role for the RE in online spatial memory retrieval rather than offline memory consolidation. However, Loureiro et al. (2012) proposed that the RE may be involved in the long-term consolidation of spatial memories. Rats trained and probed in MWM showed increased c-Fos levels in the RE/RH 25 d, but not 5 d later. Fiber-sparing excitotoxic lesions of the RE/RH did not affect acquisition or probe-trial performance at 5 d, but impaired spatial memory at 25 d. Lidocaine inactivation of the RE right before the probe trial at 25 d, however, did not affect performance. Klein et al. (2019) demonstrated that the RE is involved in remote spatial memories by affecting structural plasticity in the mPFC and HPC. RE lesions prevented the increase in dCA1 mushroom spines observed between days 5 and 25 after spatial learning. RE lesions also prevented spinogenesis in the anterior cingulate cortex, as observed 25 days after training. Ali et al. (2017) confirmed RE lesion-induced remote spatial memory impairment and further showed that environmental enrichment can alleviate this impairment. It is important to note that results from these lesion studies do not reflect RE’s individual contribution to remote spatial memories, as the RH was lesioned alongside. Overall, these results may suggest a role for the RE in the persistence of spatial memories. However, its active involvement in the consolidation and reconsolidation processes, possibly through information exchange between the mPFC and HPC during slow-wave sleep, should be further investigated by selectively inactivating the RE at distinct timepoints after spatial memory acquisition and by systematically probing the memory at those timepoints.

Barker and Warburton (2018) showed the involvement of the RE in long-term associative recognition memory using an object-in-place task. RE/RH lesions impaired long-term (3 h), but not short-term (5 min) object-in-place task performance. Non-associative recognition memory was also unaffected by these lesions. Muscimol infusion in the RE 15 min before the sample phase deteriorated object-in-place task performance at 3 h, but not at 5 min, on the delayed test, suggesting an impairment in acquisition for the long-term memory. When muscimol infusions were administered after the sample phase and before the 3 h-delay test, there was also an impairment in the retrieval. Additionally, authors revealed that protein synthesis in the RE is key for associative recognition memory. Protein synthesis inhibitor anisomycin infusions in the RE before the sample phase impaired object-in-place recognition memory at 24 h, but not at 3 h, delay.

Several reports have also examined RE’s involvement in remote fear. Wheeler et al. (2013) implicated the RE as a functional connectome with the mPFC and HPC in the retrieval of remote (36-day-old) contextual fear memories. Vetere et al. (2017) extended on these findings. Animals underwent contextual fear conditioning following chemogenetic silencing of the RE and were tested for fear memory 10 days later. Fear memory was impaired in animals treated with CNO compared to those treated with vehicle, suggesting a role for the RE in long-term contextual fear memories. Silva et al. (2018) examined brain activity patterns following a remote (30-day-old) fear memory protocol in mice. Both remote fear memory recall and extinction training increased c-Fos levels in the RE compared to homecage and no-shock controls. Silva et al. (2021) showed that RE-to-BLA projections play a critical role in the extinction of remote fear memories. Quet et al. (2020) revealed that permanent RE/RH lesions impair remote contextual fear memories without affecting recent contextual and recent or remote cued fear memories. Chemogenetically inhibiting the RE/RH did not affect recall of recent or remote fear memory recall, and contextual fear memory recall did not increase c-Fos levels in the RE, suggesting a role for the RE in contextual fear memory persistence. The RE may not always be necessary to retrieve an already established memory. It may be particularly involved when retrieval requires flexible, context-dependent regulation of fear, retrieval of extinction memories, and coordinating mPFC-HPC interactions to suppress context-inappropriate fear expression.

Troyner et al. (2018) inactivated the RE with muscimol immediately after contextual fear conditioning. This led to an impairment in the consolidation of contextual fear memory, resulting in the generalization of fear to a novel context. They also observed impairments in remote fear memory retrieval (21d), with higher freezing levels in the conditioning context and increased generalization to a novel context at this remote timepoint, revealing that the RE is critical for the consolidation of contextual fear memories. Troyner and Bertoglio (2020) briefly exposed animals to the conditioning context to reactivate fear memories, creating a space for memory reconsolidation. Post-reactivation intra-RE infusions of anisomycin and systemic injections of clonidine, an α2-adrenergic receptor agonist, impaired fear memory reconsolidation. However, pre-reactivation intra-RE infusion of muscimol blocked this effect, suggesting that the RE is critical for fear memory reconsolidation. In a follow-up study, they showed that both protein degradation and GluN2B receptors in the RE are key to this process, insofar as inhibition of protein degradation with clasto-lactacystin *β*-lactone and GluN2B receptor antagonist ifenprodil both reversed reconsolidation impairments. Troyner and Bertoglio (2021) demonstrated that RE inactivation with muscimol following a brief exposure to the conditioning context (memory reactivation) impaired conditioned freezing to that context the next day. Furthermore, GluN2A-containing glutamate receptors in the RE were critical for memory reconsolidation, as antagonism of these receptors with TCN infusions in the RE similarly impaired fear memory reconsolidation. Sierra et al. (2017) revealed that pharmacological inactivation of the anterior cingulate cortex before contextual fear conditioning causes impairments in the retrieval of remote fear memories. This memory was rescued by memory reactivation. However, inactivating the RE prior to this reactivation session reversed the effect, suggesting that the RE is key for contextual fear memory reconsolidation, possibly by mediating cortical-hippocampal communication.

Troyner et al. (2018) inactivated the RE during the fear-memory consolidation period and examined the effect on extinction memory. Although muscimol- and vehicle-treated rats both showed within-session decreases in freezing during extinction training, muscimol-treated rats exhibited higher freezing throughout the extinction session. When tested the next day, muscimol-treated animals exhibited impaired extinction to the conditioning context and increased generalization of fear in the novel context. Vasudevan et al. (2022) directly examined the role of the RE in consolidation and reconsolidation of extinction memories. They found that muscimol infusion in RE immediately after extinction training does not impair consolidation of extinction. Likewise, RE muscimol infusions immediately after reactivation of extinction memory did not impair reconsolidation of extinction. Overall, the RE may play a role in consolidation and reconsolidation of contextual fear memories, but not extinction memories. However, because few studies support the findings by Vasudevan et al. (2022), additional work is required to determine how the RE contributes to the consolidation and reconsolidation of extinction memories.

### 3.5 Higher-order cognitive processes

Based on its bidirectional connections with the mPFC, the RE has been examined for its role in cortical executive function. Dolleman-van der Weel et al. (2009) tested the effects of neurotoxic RE lesions on water maze learning. RE-lesioned rats learned to swim to the hidden platform. When tested for memory of the platform location in its absence, they initially approached the training location but did not persistently search there. Rather, they searched more generally in the pool. These results imply that the RE may have a role in behavioral strategy shifting and flexibility. In the absence of the RE, hippocampal spatial cues may not be used effectively, and animals may become impulsive, suggesting a prefrontal cortical deficit. However, Hembrook and Mair (2011) did not find any effects of RE/RH lesions on the serial visuospatial reaction time task performance, which requires prefrontal cortical activity. They proposed that RE inactivation may only impair memory in tasks that involve both cortical and hippocampal activity. Cholvin et al. (2013) used the double H water maze in which animals learned two escape strategies to find the platform. On the test day, they had to switch response strategies using memory for the place. Both mPFC and HPC muscimol inactivation impaired performance on this task, suggesting involvement of both structures. Importantly, RE/RH muscimol inactivation, too, induced strategy shifting and memory retrieval deficits. Cholvin et al. (2013) concluded that the RE may contribute to strategy shifting directly or indirectly by relaying cortical input to the HPC. Prasad et al. (2012) examined the effects of neurotoxic RE lesions on a 5-choice reaction time task, which probes attention and inhibitory control. Compared to sham controls, RE-lesioned animals did not show impaired response accuracy or latency. However, RE-lesioned animals could not inhibit premature responses while waiting for the visual cue to collect the reward. They exhibited fewer response omissions in trials and were faster at collecting rewards, consistent with impulsive behavior. On the other hand, RE-lesioned animals did not exhibit perseverative behavior, indicating two distinct effects of RE lesions on executive function: decreased inhibitory control and increased impulsivity.

Linley et al. (2016) used an odor texture discrimination task to examine the effects of RE/RH lesions on the formation of attentional sets, attentional set shifting, and reversal learning/behavioral flexibility. RE-lesioned rats showed poorer reversal learning when they had to reverse their response strategy for obtaining rewards, as well as poorer intra-dimensional shift compared to sham controls. Authors concluded that the RE may be critical for behavioral flexibility when novel situations are encountered, and that its involvement in behavioral flexibility may be due to its projections to the orbitofrontal cortex. These findings by Prasad et al. (2012) and Linley et al. (2016) implied that RE lesions are sufficient to impair PFC-dependent executive function. Viena et al. (2018) found similar impairments in behavioral flexibility during reversible inactivation of the RE in a delayed nonmatch-to-sample task. Rats were given 10 trials to correct their incorrect arm choices. RE inactivation led to significantly higher perseverative errors. Furthermore, these animals had impairments in alternating arms after correct choices. Rojas et al. (2024) demonstrated that chemogenetic inactivation of the RE impairs behavioral flexibility in the attentional set-shifting task. Chemogenetic inactivation of RE terminals in the orbitofrontal cortex impaired rule abstraction without affecting reversal learning or set shifting. Contrary to these findings, using multiple prefrontal-dependent executive function tasks, Prasad et al. (2017) revealed that RE lesions can enhance executive functions. Following RE excitotoxic lesions, the authors observed increased attention and behavioral control, decreased impulsivity, and facilitated stimulus–reward associative learning.

Overall, despite some contradictory findings, these results point to a role for RE in executive function, which can be task-specific and may also reflect the extent to which each task requires mPFC-HPC coordination. Tasks that require flexible updating of response strategies using contextual and memory-guided information may be more sensitive to RE disruption.

### 3.6 RE and stress

Cullinan et al. (1995) reported that the RE expresses moderate to high numbers of c-Fos transcripts after both swim and restraint stress. Thirty minutes after swim stress, the RE also exhibited zif-268 mRNA induction. Canteras et al. (1997) demonstrated Fos immunoreactivity in the RE following predator exposure stress. In line with these findings, Kafetzopoulos et al. (2017) showed that RE lesions or inactivation can reduce immobility in the forced swim test and can block chronic mild stress-induced dendritic length and apical dendritic spine density reductions in mPFC neurons. Moreover, RE lesions decreased serum corticosterone levels. These results indicate that RE can be an important node that responds differently to various stressors, and suppressing RE activity can alleviate stress symptoms. Kafetzopoulos et al. (2021) showed that RE lesions can also prevent chronic mild stress-induced atrophy in HPC neurons in male rats. Kafetzopoulos et al. (2024) extended RE lesion effects on the forced swim stress in female rats. For both sexes, RE lesions decreased immobility duration and increased swimming duration. However, following swim stress, RE activity, as indexed by c-Fos density, showed a sex difference, with females exhibiting significantly lower activity. The opposite pattern was observed for the mPFC and HPC activity after swim stress, with females showing higher c-Fos expression. Uliana et al. (2022) exposed male rats to a 10-day stress protocol consisting of foot shocks and restraint stress. This protocol increased immobility in the forced swim test, which was reversed by RE TTX inactivation. Following the stress protocol, there was a downregulation of spontaneously active dopaminergic cells in the VTA, a change that was similarly reversed by RE inactivation. Uliana and Grace (2025) suggested an age-dependent effect of stress on female rats. Stress during early adolescence increased the proportion of burst-firing RE neurons, whereas late adolescence stress increased the number of spontaneously active neurons. Overall, these results show a clear involvement of the RE in stress. Stress increases RE activity, and RE inactivation may buffer against stress-induced behavioral, structural, morphological, and physiological effects. Sex differences are also evident and need further research.

## 4 RE synchronization of mPFC-HPC network oscillations giving rise to emotional, memory, and behavioral processes

Previous work has emphasized that the RE is well-positioned to facilitate information exchange between the mPFC and HPC and, therefore, modulate cortical and hippocampal processes involved in memory and emotion (Bouton et al., 2021; Griffin, 2021; Ito et al., 2018; Ito et al., 2015; Plas et al., 2024; Tuna et al., 2025a; Dolleman-van der Weel et al., 2019). Several researchers have proposed different mechanisms for this modulatory role. For instance, Eichenbaum (2017) proposed that the RE may coordinate synchrony directed from the mPFC or HPC, depending on the task. To convey hippocampal contextual input to the mPFC, RE engages oscillatory synchrony from the HPC to the mPFC. Conversely, conveying cortical input to the HPC during memory retrieval requires synchrony directed from the mPFC to the HPC. Thus, the mPFC can suppress irrelevant memories and retrieve the appropriate one.

Medial prefrontal control over the HPC is in line with the retrieval suppression model, namely, suppressing unwanted, intrusive, or irrelevant memories and retrieving the appropriate memory (Anderson and Green, 2001). This type of suppression requires an inhibitory control mechanism (Anderson et al., 2016; Anderson et al., 2025). In humans, retrieval suppression is usually studied with the think/no-think paradigm in which participants study word or picture pairs. In the test phase, they are shown one item from the pair and are asked either to remember or to suppress the retrieval of the other item (Anderson and Green, 2001). Retrieval suppression trials in the think/no-think paradigm reveal functional activation in the dorsolateral and ventrolateral PFC, mostly in the right hemisphere, as revealed by neuroimaging methods such as functional magnetic resonance imaging (fMRI). The PFC activation is accompanied by activity reductions in the HPC, and the activity increase in the PFC is negatively correlated with HPC activity (Depue et al., 2010; Depue et al., 2007). Moreover, several findings showed that the PFC exerts top-down control over HPC activity during retrieval suppression (Benoit et al., 2015; Gagnepain et al., 2014).

Anderson et al. (2016) has proposed that the mPFC can control retrieval suppression by two routes: the entorhinal cortex (the entorhinal gating hypothesis) and the RE (the thalamo-hippocampal modulation hypothesis). The PFC projects to the HPC either through the entorhinal cortex or the RE. Alternatively, both the RE and entorhinal cortex may be involved in retrieval suppression, although at different times. The entorhinal cortex may be involved proactively, before the retrieval process starts, to gate the cue input that initiates it. The RE, on the other hand, may involve reactively, if the entorhinal gating of suppression fails and the retrieval has already started. Both hypotheses are supported by imaging findings, although each has several shortcomings. See Anderson et al. (2016) for a more detailed discussion.

The RE may facilitate information exchange between the mPFC and HPC by synchronizing network oscillations (Angulo-Garcia et al., 2020; Cassel et al., 2021; Eichenbaum, 2017; Ferraris et al., 2018; Hauer et al., 2019; Hauer et al., 2022; Plas et al., 2024; Totty and Maren, 2022). Oscillations provide a means of communication between brain regions, including those that are distant from each other, by synchronizing their activity (Fries, 2015; Totty and Maren, 2022). For instance, numerous studies have revealed synchronized theta oscillations (4–12 Hz) in the mPFC and HPC during distinct tasks (Colgin, 2011; Hyman et al., 2010; Jones and Wilson, 2005; Lesting et al., 2013; Lesting et al., 2011; Mourão et al., 2026; O'Neill et al., 2013; Siapas et al., 2005; Stout et al., 2024; Totty et al., 2023). Lesting et al. (2011) demonstrated IL-dHPC theta synchrony during extinction retrieval in mice. Moreover, IL theta led HPC theta during extinction retrieval but not after extinction training (Lesting et al., 2013). These results are in line with models suggesting that mPFC control over HPC underlies memory retrieval (Anderson et al., 2016; Anderson et al., 2025; Eichenbaum, 2017). mPFC-HPC theta coupling, with mPFC theta leading HPC theta, might be a mechanism for this and also underlying extinction retrieval. Later, confirming Lesting et al. (2011) results, Totty et al. (2023) revealed mPFC-dHPC 6–9 Hz theta coupling during extinction retrieval in rats. Moreover, theta synchrony was observed for both PL-HPC and IL-HPC pairs.

mPFC-HPC oscillatory synchrony across different memory tasks laid the groundwork for studies examining the RE’s involvement in this synchrony. Hallock et al. (2016) revealed a causal role for the RE in mPFC-HPC theta synchrony during a spatial working memory task in rats. mPFC and dHPC showed theta synchrony during this task, which was abolished with muscimol infusions in the RE. Kafetzopoulos et al. (2017) recorded LFPs in the PL and vCA1 in anesthetized rats. RE lesioned animals did not show reduced activity in the PL and vCA1 but did show reduced mPFC-vHPC delta and theta coupling compared to sham-operated rats. Ito et al. (2018) observed increased theta-spike-time coordination in the mPFC-RE-HPC network in a T-maze alternation task before choice points. Specifically, mPFC and RE neuronal spiking showed coherence with HPC theta oscillations. Ferraris et al. (2018) revealed that RE muscimol inactivation abolishes mPFC-HPC gamma synchrony. Malsen et al. (2023) demonstrated mPFC-RE-vHPC beta coherence during the choice phase in a working memory task. The RE was not involved in the increase in mPFC-vHPC theta coherence during working memory. The authors proposed that beta oscillations may be critical for top-down mPFC influence over the HPC for executive control, while theta and gamma oscillations may provide bottom-up information to the mPFC. Stout et al. (2022) employed an HPC-dependent delayed alternation task to examine the role of the RE in vicarious trial and errors. RE inactivation increased such errors and perseveration, decreased mPFC-HPC theta coherence, and disrupted mPFC-to-HPC theta directionality during vicarious trial and errors compared to non-error trials. Later, Stout et al. (2024) used a closed-loop brain-machine interface to manipulate trials in a similar delayed alternation task, based on mPFC-HPC theta synchrony magnitude. Trials that started during high theta synchrony led to a higher percentage of correct choices than those started during low theta synchrony. mPFC-RE interactions were particularly sensitive to mPFC-HPC oscillatory synchrony, with strong mPFC-HPC synchrony also leading to strong mPFC-RE synchrony, and mPFC theta oscillations leading to RE oscillations. Furthermore, theta-paced optogenetic stimulation of the RE invariably created theta power in the mPFC. RE stimulation at around 8 Hz led to variable mPFC-HPC theta coherence patterns, depending on the current HPC oscillatory activity. Recently, Basha et al. (2025) performed recordings from the mPFC-RE-HPC network in cats during sleep. They revealed a dynamic and oscillation-dependent dialogue between the mPFC and RE. Overall, these results show that the RE facilitates mPFC-HPC oscillatory synchrony in different frequency bands, possibly depending on the task.

Totty et al. (2023) demonstrated that the RE coordinates oscillatory synchrony in the mPFC and HPC during extinction retrieval. Initially, Totty et al. (2023) found that extinction retrieval is characterized by increased 6–9 Hz theta oscillatory power in both the mPFC and HPC. Moreover, the two structures displayed oscillatory coherence at 6–9 Hz. The RE had similar 6–9 Hz power following extinction learning. Totty et al. (2023) then recorded LFP activity from the mPFC and dHPC while pharmacologically inactivating the RE. RE inactivation prior to the extinction retrieval test impaired both the mPFC-dHPC theta oscillatory coherence and the retrieval of extinction memories. Thus, RE plays an important part in mPFC-HPC network theta synchrony, which also supports extinction retrieval. In line with this, optogenetic theta-paced 8 Hz stimulation of the RE blocked fear renewal (Totty et al., 2023), a common type of fear relapse occurring when extinguished CSs are encountered outside the extinction context (Bouton, 1993; Bouton and Ricker, 1994; Maren et al., 2013). Totty et al. (2023) revealed that 8 Hz RE stimulation reliably creates dominant 8 Hz power in the RE, with RE neurons entrained to photo-stimulation. Complementing these findings, Tuna et al. (2025a) optogenetically stimulated the RE at 8 Hz, while simultaneously recording LFPs from the mPFC and dHPC. RE optogenetic stimulation during extinction facilitated the retrieval of extinction memories the next day, in a stimulation-free retrieval test. Theta power in the RE may help facilitate extinction memory and suppress fear, possibly by synchronizing the mPFC and HPC. Indeed, Tuna et al. (2025a) demonstrated that 8 Hz RE stimulation drives 8 Hz power in both the mPFC and dHPC separately while also increasing the oscillatory coherence between the two at this stimulation frequency.

Some studies reported that the RE has a minimal role in synchronizing mPFC-HPC theta oscillations. Roy et al. (2017) recorded LFPs from the PFC, HPC, and RE in anesthetized rats. RE pharmacological inactivation did not significantly affect PFC-HPC theta coherence. Jayachandran et al. (2023) reported mPFC-HPC beta coherence during a non-spatial sequence memory task. RE beta preceded mPFC and HPC beta, which implies that RE beta can drive the mPFC and HPC. mPFC-HPC theta coherence, on the other hand, appeared during non-memory-related running. Optogenetic RE activation increased mPFC-HPC beta coherence while decreasing theta coherence. The authors concluded that RE facilitates mPFC-HPC interaction during memory-based decision- making by synchronizing beta activity within this network. Malsen et al. (2023) also reported beta coherence in the mPFC-RE-vHPC network during a touch screen-based working memory task. These discrepancies may be partly explained by differences in the behavioral and memory tasks used across studies, as well as methodological differences in recording conditions, including whether recordings were performed in awake or anesthetized animals.

Overall, the RE contributes to various memory processes, such as extinction retrieval, memory-guided decision-making, and spatial working memory. As detailed above, there is now considerable evidence that the RE achieves this by synchronizing mPFC-HPC oscillations. Particularly, for memory retrieval, including extinction retrieval, RE-mediated theta synchrony may facilitate mPFC top-down control over HPC-dependent memories. In other contexts, beta or gamma synchrony may support distinct forms of memory-guided decision-making and executive control. Thus, the RE may flexibly organize mPFC-HPC communication in response to ongoing task requirements. In line with its bidirectional connections with the mPFC and HPC, the direction of oscillations may also change dynamically, depending on task demands.

## 5 RE and neuropsychiatric, neurological, and neurodegenerative disorders

Just as the RE-mediated mPFC-HPC communication supports adaptive context- and task-dependent behavior, disruptions in RE function may give rise to maladaptive circuit dynamics that contribute to neuropsychiatric, neurological, and neurodegenerative disorders. In this section, we review work implicating the RE in these disorders.

### 5.1 Schizophrenia

Schizophrenia is known to disrupt mPFC and HPC function and synchrony (Hill et al., 2004; Joyce et al., 2002; Lynall et al., 2010; Pratt et al., 2017; Sigurdsson et al., 2010; Tamminga et al., 2010). It particularly reduces connectivity in thalamocortical circuits (Anticevic et al., 2014; Giraldo-Chica and Woodward, 2017; Tu et al., 2019; Welsh et al., 2010; Woodward et al., 2012). The RE, therefore, is likely to be affected by schizophrenia.

Increased delta oscillations in thalamocortical circuits are commonly observed in waking states in schizophrenia (Boutros et al., 2008; Siekmeier and Stufflebeam, 2010; Sponheim et al., 1994). As schizophrenia leads to working memory impairments and the RE is known to be involved in working memory, several researchers have examined how delta power in the RE interacts with working memory performance. For example, delta-paced optogenetic stimulation of RE terminals in the dCA1 impaired working memory in a delayed alternation task (Duan et al., 2015; Rahman et al., 2021), an effect observed in both rats and mice.

NMDA receptor antagonism can mimic behavioral symptoms of schizophrenia, and increased delta oscillations are observed in schizophrenia (Abi-Saab et al., 1998; Coyle, 1996; Olney et al., 1999). Goswamee et al. (2021) confirmed spatial working memory deficits after acute treatment with NMDAR antagonist ketamine, which increased glutamate release from the mPFC in the RE, and also increased the firing of RE neurons, which can then innervate the HPC. Zhang et al. (2009) revealed that NMDAR antagonism with APV in the reticular nucleus causes delta frequency bursting. Furthermore, they showed that NMDAR antagonists act synergistically with dopamine to produce delta bursting and that D2 receptor antagonists abolished delta bursting. The reticular nucleus is bidirectionally connected with the RE (Çavdar et al., 2008). It can drive RE activity in the presence of NMDAR antagonists, which may, in turn, innervate and activate the HPC. Väisänen et al. (2004) reported that treatment with the systemic NMDAR antagonist, MK801, increases activity in the RE and CA1. They also showed that ketamine treatment increases dopamine release. NMDAR antagonist-evoked increases in dopaminergic cell activity were not driven by direct effects of antagonists on dopaminergic cells. Rather, glutamatergic projections to these cells were critical (Zhang et al., 1992). Several reports have shown that hippocampal activity leads to dopamine release (Floresco et al., 2001; Legault et al., 2000; Luo et al., 2011). Indeed, the dopamine system structures are not disrupted in schizophrenia. Rather, dopamine system hyperresponsivity may be due to afferents, such as the HPC (Grace, 2016; Lodge and Grace, 2007). Based on these pieces of evidence, Lisman et al. (2010) proposed a thalamo-hippocampal-VTA loop that can explain the high basal activity in the thalamus and dopamine system in schizophrenia. Namely, dopamine hyperpolarizes the reticular nucleus and works synergistically with the NMDA system (Zhang et al., 2009), giving rise to delta bursting in the reticular nucleus, which activates the RE, which then innervates the HPC. HPC activity, in turn, leads to more dopamine release. Through this positive feedback loop, the RE may have a key role in functional and behavioral phenomena reported in schizophrenia. Similarly, Zimmerman and Grace (2016) showed that the mPFC-RE-ventral subiculum pathway controls ventral tegmental area dopamine neuron firing. Disruptions in this pathway may lead to a hyperdopaminergic state, which may play a role in schizophrenia.

Subsequently, Zhang et al. (2012) provided more direct evidence on the effects of NMDA antagonism in the RE and HPC. They observed increased firing rate and delta power in both the RE and CA1 following systemic ketamine injections. The RE and CA1 showed increased delta-frequency coherence, and spikes in both these regions were modulated by delta oscillation. RE muscimol infusions suppressed ketamine-induced delta power in the HPC, whereas RE ketamine infusions increased hippocampal delta power, suggesting that the delta oscillation in the HPC is the result of RE projections rather than local actions of ketamine in this structure. Overall, disruption of the thalamo-cortico-hippocampal synchrony may bias network activity toward maladaptive oscillatory states, such as excessive delta coupling, thereby contributing to cognitive dysfunction and hyperdopaminergic activity. Thus, the RE may represent a critical node through which disturbances in mPFC-HPC communication are translated into the circuit-level and behavioral abnormalities associated with schizophrenia.

### 5.2 Epilepsy

Considerable work points to a role for the RE in epilepsy. Cavalheiro et al. (1987) observed morphological damage in RE neurons following pilocarpine-induced status epilepticus. Peredery et al. (2000) induced status epilepticus with a single systemic injection of lithium chloride and pilocarpine, and reported degeneration of neuronal populations in the RE. Scholl et al. (2013) proposed that the RE may be one of the structures involved in epileptogenesis. The lithium pilocarpine model of acquired epilepsy in rat pups revealed severe degeneration in RE neurons. Montpied et al. (1995) reported lower calbindin levels in the RE in two strains of genetically epilepsy-prone rats. Lower RE calbindin levels may alter calcium-buffering capacity and calcium-mediated processes, leading to seizures. Drexel et al. (2011) demonstrated loss of calretinin neurons in the RE and calretinin fibers in the subiculum and entorhinal cortex, efferent structures of the RE, in epileptic rats.

Using midline thalamic lesions in cats, Hiyoshi and Wada (1988) showed that the RE participates in amygdala-kindling but is not involved in the maintenance of amygdala-kindled seizures. Hirayasu and Wada (1992a) reported that NMDARs in the RE are involved in convulsive seizures in rats: NMDA injections in the RE caused seizures, with fatal status epilepticus in some cases. Hirayasu and Wada (1992b) also investigated the effect of NMDA injections into the RE on amygdala- and HPC-kindling in rats. For both amygdala- and HPC-kindled animals, NMDA injections in the RE required less stimulation for stage five seizures to develop compared to injections outside of the RE.

Bertram et al. (1998) revealed that thalamic stimulation in the vicinity of RE causes significant epileptiform responses in CA1 of epileptic, but not control, rats. Increased population spikes following such ventral midline thalamic stimulation were comparable to those observed following CA3 stimulations. Bertram et al. (1998) thus suggested that midline thalamic nuclei are part of the limbic seizure network. Subsequently, Bertram et al. (2001) showed that seizures evoked by HPC-kindling stimulations activated the midline thalamus, with the thalamic activity increasing from the initiation of stimulation and showing a parallel pattern to that of hippocampal activity. This may indicate early involvement of the midline thalamus in seizure activity, before the motor seizure behavior starts. The midline thalamus was also active and showed parallel hippocampal activity in chronically epileptic animals. Anterior and posterior RE had significantly lower cell numbers in epileptic animals than in kindled and control animals, suggesting that cell loss may be due to the epileptic condition rather than experiencing seizures. Furthermore, the authors revealed that RE inactivation with lidocaine can shorten after-discharges in CA1 and CA3-kindled rats, supporting that RE activity is critical for limbic seizure activity in the HPC.

Aracri et al. (2018) performed simultaneous *in vitro* recordings from the CA1 and RE in guinea pig brains. They induced seizures via arterial perfusions of GABAAR antagonist bicuculline. The RE showed seizure-like events following bicuculline perfusions, indicating its involvement from early stages. CA1 led bursts in seizure-like events. However, in the final stages of seizure-like events, the CA1 burst lead shifted to the RE. More recently, Dabrowska et al. (2019) evoked seizures via electrical stimulation of the HPC in TRAP mice, and TRAPed neurons were examined following tissue clearing. The RE showed increased activity at the peak of status epilepticus. Sloan and Bertram (2009) stimulated the RE and recorded responses from the PFC in epileptic, kindled, and control rats. There was a significant reduction in RE stimulation-evoked responses in the PFC of epileptic rats, suggesting an impairment in the RE-to-PFC connection. Authors observed cell loss in the RE but not the PFC, which may explain the attenuation of the thalamically evoked PFC response. Finally, in three human participants, Romeo et al. (2019) showed the involvement of the midline thalamus in epilepsy: the midline thalamus was recruited during seizure initiation and propagation, and electrical stimulation of the thalamus evoked seizures.

Overall, these findings place the RE among structures undergoing neuronal degeneration in epilepsy and suggest that it plays a role in epilepsy, potentially via its distributed connections with the HPC, amygdala, mPFC, subiculum, and entorhinal cortex. It may be a critical hub through which abnormal activity can propagate across limbic and prefrontal-hippocampal networks. Given its anatomical position between the mPFC and HPC, disruptions in RE function may impair the normal coordination of mPFC-HPC oscillatory activity and instead promote pathological synchronization within the broader limbic seizure network. More work is needed to reveal the mechanism by which the RE impacts seizures. For a more detailed review of the RE’s role in epilepsy, also see Dolleman-van der Weel and Witter (2020).

### 5.3 Depression

Kafetzopoulos et al. (2017) reported that the forced swim test increases c-Fos levels in the RE. Furthermore, RE-lesioned male rats demonstrated reduced immobility in the forced swim test compared to sham-operated animals. Immobility levels in the RE-lesioned rats were comparable to those of the selective serotonin reuptake inhibitor sertraline-treated positive controls, suggesting that RE inactivation can mimic antidepressant-like behavioral responses. Transient RE inactivation with tetracaine led to similarly lower immobility levels. RE lesions prior to the chronic mild stress model of depression prevented the decreased sucrose preference that is caused by this model. Moreover, similar to the effects of sertraline treatment, RE-lesioned rats did not have atrophy in the PFC neuron dendrites following the chronic mild stress model of depression. The authors concluded that disrupting the RE function blocks depressive-like behaviors associated with the chronic mild stress model of depression. However, antidepressant-like effects of RE lesions were observed when the lesions occurred before the animals underwent the chronic mild stress, suggesting that RE lesions can prevent but not attenuate the effects of chronic mild stress. Kafetzopoulos et al. (2024) extended these findings to female rats. RE lesions induced antidepressant-like effects in both male and female rats. Sertraline treatment further increased swimming duration and decreased immobility duration in RE-lesioned rats compared to sham controls in both sexes. Treatment with a tricyclic antidepressant, clomipramine, increased climbing behavior.

Kafetzopoulos et al. (2024) also reported differential mPFC, RE, and HPC activity across sexes following antidepressant treatment. After forced swim stress, female rats showed higher c-Fos expression in the mPFC and HPC and lower c-Fos expression in the RE with both vehicle and sertraline treatments. For a detailed review on RE and sex differences, also see Cassel et al. (2025). Bao et al. (2025) reported that RE activation or activation of the RE excitatory projection to the mPFC may induce depressive-like behaviors in male mice. On the other hand, inhibition of this projection alleviated hyperalgesia and depression-like behaviors in neuropathic pain mice. Based on these findings, the authors proposed a role for the RE in pain sensation and associated emotional aspects, including depression. Therefore, the RE may be involved in depression, with its inactivation being protective from depression-like behaviors and dendritic atrophy. Stress and depression may involve maladaptive or excessive coordination of the mPFC-HPC circuit. RE inactivation may be protective by preventing pathological engagement of this circuit under stress. Therefore, RE synchronization of the mPFC-HPC network may lead to divergent outcomes depending on stress history, including facilitating maladaptive affective and neuroplastic changes under chronic stress. Future research should explicitly test whether depressive-like behaviors are associated with maladaptive RE-mediated synchronization of mPFC-HPC circuits.

### 5.4 Fetal alcohol spectrum disorder

There is a growing interest in the role of midline thalamic nuclei in substance use disorders, particularly given their projections to the mPFC, HPC, and nucleus accumbens. So far, evidence for the involvement of the RE in addiction has been suggestive, but causal mechanisms are not yet definitive. Jaramillo et al. (2016) showed that the RH modulates sensitivity to alcohol, even though they did not selectively study the RE in this work. Gursky et al. (2019) examined damage to the RE in a rodent model of binge drinking during the third trimester of human pregnancy. Female rat pups with alcohol exposure in postnatal days 4–9 showed a reduction in total neuron number, but not non-neuronal cell number, in the RE in adulthood. There was also a reduction in RE volume. Strikingly, these effects were specific to the RE, and no reduction in cell number or volume was observed in the RH. A lower dose of alcohol than what is used by Gursky et al. (2019) was also capable of causing these effects (Gursky and Klintsova, 2022). Using a similar alcohol exposure paradigm, researchers reported an increase in the number of RE neurons projecting to both the mPFC and vHPC in alcohol-exposed male and female rats, whereas the number of mPFC- or vHPC-projecting RE neurons was unaffected (Gursky and Klintsova, 2021). Although the functional implications of this increase are not clear, the authors emphasized that an increased or hyperactive mPFC-RE-vHPC connection may not be adaptive. The researchers also reported a reduction in the length of mPFC axon terminals in the RE (Smith et al., 2022), and performance deficits in the object-in-place task (Smith et al., 2025) in alcohol-exposed rats. Moreover, both effects were more pronounced in females, revealing sex differences in the effects of alcohol on RE function. Critically, a recent work by Rosenblum et al. (2025) demonstrated altered choice behavior, decreased mPFC theta oscillations, increased HPC beta oscillations, and decreased mPFC-HPC theta coupling in alcohol exposed rats. Although the contribution of the RE to these oscillatory changes was not directly tested, the reported decrease in mPFC-HPC theta coupling is notable. Alcohol-induced alterations in RE structure and connectivity may contribute to disrupted prefrontal-hippocampal oscillatory synchrony and associated cognitive deficits. It is also important to note that studies summarized in this section used the same alcohol exposure protocol. There is clearly a need for an in-depth understanding of the role of RE in addiction, using different protocols and substances. For a detailed review on alcohol-use disorder-related damage in midline thalamic nuclei, also see Savage et al. (2020).

### 5.5 Obsessive-compulsive disorder

Studies examining the role of the RE in obsessive-compulsive disorder (OCD) are scarce. Prasad et al. (2012) demonstrated that RE lesions elicit impulsive but not compulsive responses in an inhibitory control task. Goh et al. (2025) showed that optogenetic stimulation of the RE causes excessive, repetitive grooming, which can interrupt and reduce ongoing drinking and eating behavior in water and food-deprived rats, respectively. RE photo-inhibition, on the other hand, ceased the maintenance of grooming behavior. Goh et al. (2025) reported that RE photostimulation was aversive, as rats avoided the stimulation-paired compartment during a real-time place preference test. RE photo-stimulation also decreased time spent in the center in an open field test and in open arms in an elevated plus maze test, indicating anxiety-like behavior. Authors emphasized the similarity between compulsive grooming in rats and compulsive hand-washing in OCD patients. They also drew parallels between stimulation-evoked anxiety in rats and anxiety reported by OCD patients following their compulsive behavior. Finally, Goh et al. (2025) showed that selective optogenetic stimulation of RE terminals in the dorsal premammillary nucleus evokes persistent grooming behavior, suggesting that this thalamo-hypothalamic circuit may be involved in compulsive behavior. Later, Tuna et al. (2025a) confirmed RE photo-stimulation-evoked grooming behavior in rats. However, they postulated that RE photo-stimulation decreases fear and facilitates extinction rather than being aversive or anxiogenic. Lee et al. (2025) also reported grooming behavior with optogenetic stimulation of the RE. They proposed that RE activity is congruent with sleep need, and RE stimulation-evoked grooming may be among sleep-preparatory behaviors. Although direct evidence linking RE-mediated mPFC-HPC synchrony to OCD-like behaviors remains limited, disruptions in this coordination may contribute to the persistence of repetitive behaviors when flexible updating of behavioral responses is required. Future work should determine whether compulsive-like behaviors are associated with altered RE-dependent synchronization of mPFC-HPC circuits.

### 5.6 Alzheimer’s disease

Alzheimer’s patients show a loss of pyramidal neurons in the mPFC and HPC. Alzheimer’s disease also causes impairments in memory and executive function (Karki and Moustafa, 2021; Minter et al., 2016). The RE, therefore, is likely to be involved in Alzheimer’s disease. Previously, human neuroimaging studies revealed volume reduction and atrophy in thalamic nuclei in Alzheimer’s patients (de Jong et al., 2008; Zarei et al., 2010). Zidan et al. (2019) proposed that thalamic volume reduction can be a predictor of poor cognitive performance. Censi et al. (2024) compared volume differences of thalamic nuclei in individuals with Alzheimer’s, mild cognitive impairment, and healthy controls. Alzheimer’s and mild cognitive impairment patients who convert to Alzheimer’s showed overall thalamic atrophy compared to stable mild cognitive impairment patients and healthy controls, which did not differ from each other. Importantly, the RE underwent significant atrophy in mild cognitive impairment patients who converted to Alzheimer’s compared to those who were stable, suggesting that RE atrophy can predict conversion to Alzheimer’s.

German et al. (1987) reported neurofibrillary tangles in the RE of brains from Alzheimer’s patients, which was later confirmed by Braak and Braak (1991). Walsh et al. (2020) examined the neurophysiological changes the RE undergoes in the J20 mouse model of amyloidopathy. Using *in vitro* whole-cell patch-clamp recordings, they reported hyperpolarized membrane potentials in J20 mice compared to controls. J20 mice RE neurons were more likely to show increased rebound firing following hyperpolarizing current stimuli. Furthermore, action potential waveforms were altered with a reduction in spike width. These results indicate altered intrinsic properties in RE neurons, which may contribute to the disease. Shoob et al. (2023), using the familial Alzheimer’s disease mouse model, showed that anesthesia induces hyperexcitability in both the CA1 and mPFC, disrupts CA1 firing rate homeostasis, impairs RE-CA1 short-term facilitation, and working memory. Longitudinal (from prodromal to symptomatic stage) tonic deep-brain stimulation of the RE prevented the CA1 abnormalities and cognitive decline later during the symptomatic stage. The authors concluded that the RE can be a critical node for resilience to Alzheimer’s disease. Together, these findings suggest that Alzheimer’s disease may compromise the RE both structurally and physiologically, potentially disrupting its ability to coordinate mPFC-HPC communication required for memory and executive function. Conversely, the protective effects of RE stimulation may indicate that restoring RE-mediated mPFC-HPC interactions facilitates circuit function and cognitive performance in Alzheimer’s disease. It should also be noted that rodent findings come mostly from mouse Alzheimer’s models. Future work should explore whether the RE is similarly involved in Alzheimer’s in rats.

## 6 Conclusion

The RE, a critical hub in the mPFC-HPC network, has been the focus of extensive research on emotional and spatial processing, memory-guided behavior, and executive function. Alterations and abnormalities have also been linked to several neuropsychiatric, neurological, and neurodegenerative disorders. Here, we reviewed studies demonstrating the involvement of the RE in these processes and proposed that RE synchronization of mPFC-HPC network oscillations underlies its role. The RE may dynamically synchronize mPFC-HPC oscillations across frequency bands, such as delta, theta, or beta, in response to ongoing affective, spatial, and memory requirements. Thus, the RE may coordinate the expression of adaptive behavior by facilitating communication between the HPC and mPFC. Conversely, alterations in RE-mediated mPFC-HPC communication and network oscillations may underlie disorders such as schizophrenia and epilepsy. Thus, the RE may represent a potential therapeutic target for such disorders. More work is clearly needed to elucidate the exact mechanism by which the RE achieves these processes. RE’s function in the human brain and potential sex differences also await further research.
